# Insights into dental age estimation: introducing multiple regression data from a Black South African population on modified gustafson’s criteria

**DOI:** 10.1007/s00414-024-03312-1

**Published:** 2024-08-22

**Authors:** Fabian Rudolphi, Laurin Steffens, Denys Shay, Chané Smit, Liam Robinson, Herman Bernitz, Andreas Schmeling, Maximilian Timme

**Affiliations:** 1 Institute of Legal Medicine, University Hospital Münster, Röntgenstraße 23, 48149 Münster, Germany; 2 Department of Epidemiology, Harvard T.H. Chan School of Public Health, 677 Huntington Ave, Boston, MA 02115 USA; 3 Department of Oral and Maxillofacial Pathology, Faculty of Health Sciences, University of Pretoria, Gauteng, South Africa

**Keywords:** Age assessment, Legal medicine, Orthopantomogram, Chronological age, Premolars, Demirjian

## Abstract

Dental Age Estimation (DAE) is an effective instrument of the rule of law for verifying dubious age claims in living individuals. Once tooth development is complete, only degenerative dental characteristics can be used for this purpose. The influence of ethnicity on these degenerative dental characteristics has not been clarified.

Degenerative changes were examined using modified Gustafson’s criteria including secondary dentin formation, cementum apposition, periodontal recession and attrition using the Olze et al. (2012) staging scales. Orthopantomograms of 1882 black South Africans, consisting of 934 females and 948 males, from 12.00 to 40.96 years of chronological age were utilized. Two independent examiners performed the evaluations, with one of the two evaluating all radiographs twice.

The relationship between individual characteristics and chronological age was analyzed using multiple regression analysis with chronological age as the dependent variable. The resulting R^2^ values ranged from 0.22 to 0.35, and the standard error of estimate were between 6.6 and 7.3 years. The correlation with age was consistently lower for females compared to males. The characteristic of cementum apposition emerged as critical in this population, due to a particularly low correlation with age and observer agreements partly in the “slight” range. The formula’s values for the correlation with age were in general below the literature values for other populations. Overall, the limited precision of the age estimation by the formulae presented, especially for females, must be emphasized. The question of whether ethnicity per se exerts an influence on the characteristics in question, or whether the different socio-economic status, which encompasses factors such as nutrition and healthcare, is the determining factor, needs to be assessed in future studies.

## Introduction

Forensic age estimation in living individuals has emerged as a critical area of research within the forensic sciences, addressing legal matters related to criminal liability, immigration, and competitive sports [1–9]. Legal thresholds, such as the ages of 14, 18, and 21 years, are of significant importance in many jurisdictions, necessitating reliable methods for determining whether individuals have reached these ages [5, 10]. The Study Group on Forensic Age Diagnostics (AGFAD) recommends a multifaceted approach for forensic age estimation in living juveniles and young adults [11]. This includes a physical examination, a radiographic assessment of the left hand, and a detailed dental examination with orthopantomograms (OPGs). When hand ossification is complete, additional radiological examinations of the clavicles using conventional radiography or computed tomography are suggested [11]. When age-associated dental characteristics are used to determine a person’s chronological age, this is termed as Dental Age Estimation (DAE) [12–15].

The mineralization and eruption of the third molar are established criteria in forensic odontology for estimating age in the relevant age groups [5, 11, 16]. The topic of proof of majority by means of DAE has been the subject of extensive research. In 2016, for example, Cavrić et al. presented a promising approach for a black African population from Botswana using the third molar maturity index (I3M) [16]. However, reliance on the mineralization and eruption status of the third molars alone may often be insufficient for determining whether an individual has reached the age of 18. This is because third molar development and thus tooth development as a whole can be completed before the age of 18 [17]. Consequently, there is a pressing need for methods to estimate age after the completion of third molar mineralization, and thus DAE methods to prove that an individual has reached the age of 18 [18].

Several studies have investigated the potential use of regressive dental changes to estimate age, focusing on the period following the completion of dental development [19–26]. The seminal work of Gustafson (1947) established the scientific basis for dental age estimation based on degenerative dental changes [27, 28]. This was achieved by identifying six characteristics—secondary dentin formation, periodontal recession, attrition, apical translucency, cementum apposition, and external root resorption—that correlate with chronological age. Furthermore, Matsikidis demonstrated in 1981 that these regressive changes, initially evaluated in extracted and ground teeth, could also be assessed using basic dental radiographs [29]. Consequently, Matsikidis effectively facilitated the application of the Gustafson’s criteria to living individuals [29, 30]. However, it proved impossible to transfer the characteristics of apical root translucency and external root resorption to X-ray technology. As a result, these characteristics play a subordinate role in the age assessment of living persons today.

Researchers have refined these methods in recent years to improve their applicability to living individuals. Olze et al. (2012) modified Gustafson’s criteria for use with OPGs, developing regression formulae specifically for age estimation in individuals aged 15 to 40 [31]. In particular, Olze et al. are credited with developing unique staging scales for assessing the characteristics on an OPG [31]. Subsequent studies, including those by Timme et al. in 2017 and Si et al. in 2019, have validated the reliability of this approach across broader age ranges or different ethnic groups, respectively [32–36].

The main difference between the regressive tooth characteristics and the characteristics of the DAE, which are based on tooth development, is that tooth development is essentially genetically determined and can only be influenced by external factors within certain limits [34–37]. In contrast, the regressive characteristics may be influenced by external factors such as diet, medication, general state of health or habits [38–40]. In fact, the transition from an age-associated physiologically degenerative process to a pathological change is ultimately fluid. This makes the evaluation of regressive tooth characteristics a more significant challenge for the examiner, as pathologically altered teeth must not be used for DAE because in these cases, the age correlation is likely significantly skewed, weakened or eliminated. Furthermore, whether ethnicity influences the Gustafson criteria has not yet been conclusively clarified [32].

The present study addresses the question of the influence of ethnicity on degenerative dental characteristics by analyzing the Gustafson’s criteria on OPGs in a population of black individuals from Sub-Saharan Africa. Our study also responds to a direct need in the literature to test the method on Africans [33].

The primary research question is whether the method is suitable for reliable age estimation in the relevant age group in this population. To this end, unique regression models were developed for this population, and their goodness of fit was compared with those known from the literature for other populations. In addition to this specific question, the data obtained must be analyzed in combination with other studies to clarify the influence of ethnicity on the characteristics.

## Materials and methods

The relevant ethics committees approved the study. Firstly, approval was obtained from the Ethics Committee of the Medical Association of Westphalia-Lippe and the University of Münster (AZ 2020-038-f-S) as the study site. Secondly, approval from the Research Ethics Committee of the University of Pretoria, Faculty of Health Sciences (reference no. 587/2021) as the source of the radiographs was obtained.

The OPGs used in this study were obtained from the University of Pretoria Oral Health Centre in Pretoria, Gauteng, South Africa. These OPGs were originally taken for dental diagnostic and treatment purposes. For this study, OPGs were randomly selected from the available pool for retrospective, blinded analysis. The selection was stratified by individual years of age within the 12 to 40-year range. Subjects were categorized by their exact age, for instance, those aged between 15.00 and 15.99 years were classified as “15 years”. Inclusion criteria required that the exact age of each subject at the time of radiographic examination be known. In South Africa, patients under 16 must present a state-issued birth certificate upon admission to the dental clinic, while individuals over 16 must present an identification card.

The study exclusively involved self-identified black individuals.

South Africa is classified as an upper-middle-income country based on gross national income (GNI) [41], and therefore, this classification does not accurately reflect the socio-economic status of many South Africans, particularly those in impoverished regions. The socio-economic status of black South Africans is generally lower than the national average [42]. Furthermore, public healthcare facilities in South Africa are predominantly utilized by individuals from lower socio-economic backgrounds [43]. Consequently, the socio-economic status of the study population is below the national average and does not align with the upper-middle-income classification of the country as a whole.

The initial step involved verifying the sufficiency of the image quality. The exclusion criteria encompassed problems related to artifacts or misalignments occurring during image acquisition, resulting in distortions. During the initial selection of images from the clinic’s available pool, careful consideration was given to ensure that all lower premolars were present and free of significant pathologies, including tooth impaction, extensive destruction, or large apical lesions. Only a single OPG from each individual was considered for inclusion.

**Fig. 1:**
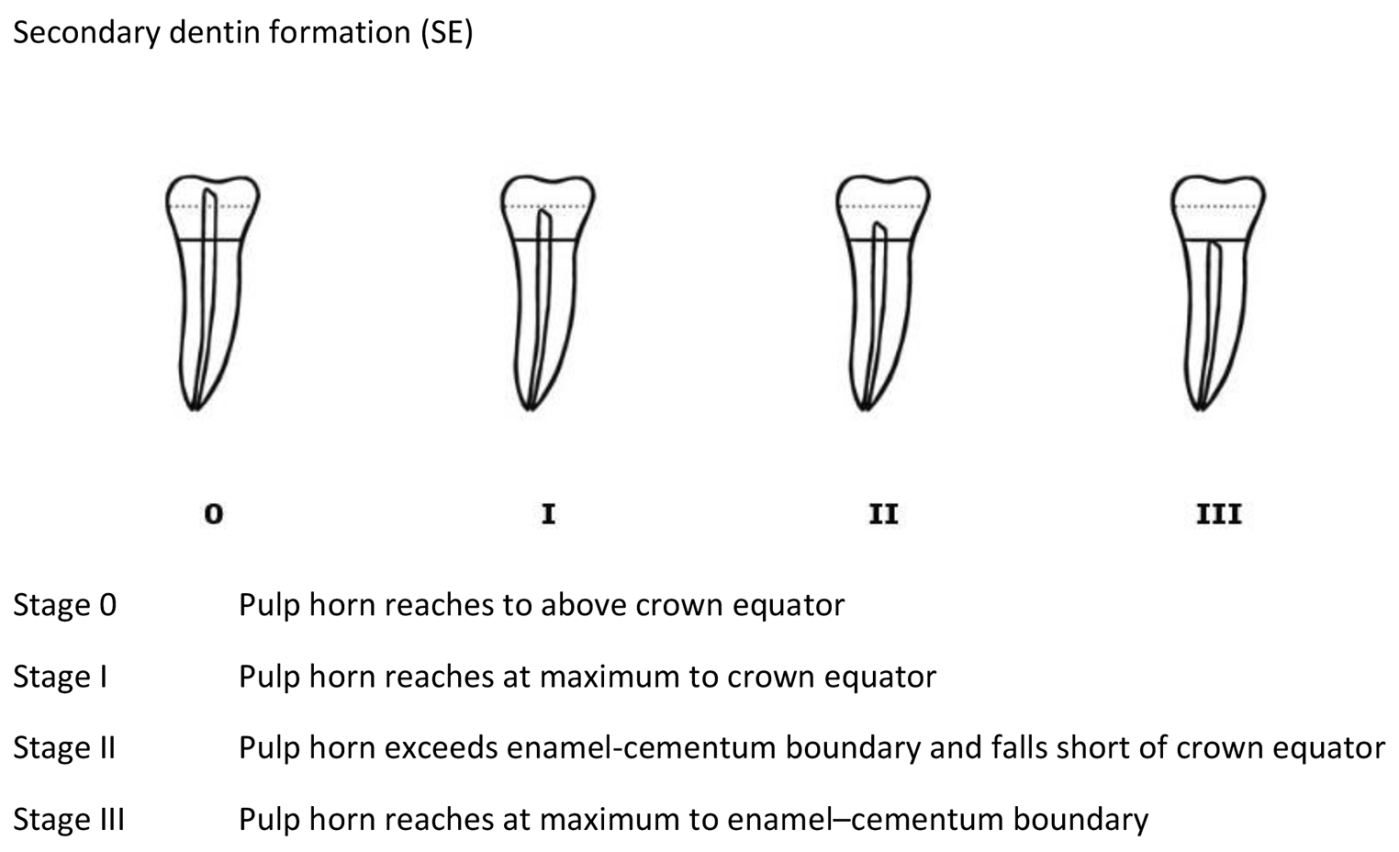
Stage classification to determine degree of secondary dentin formation [31]

**Fig. 2:**
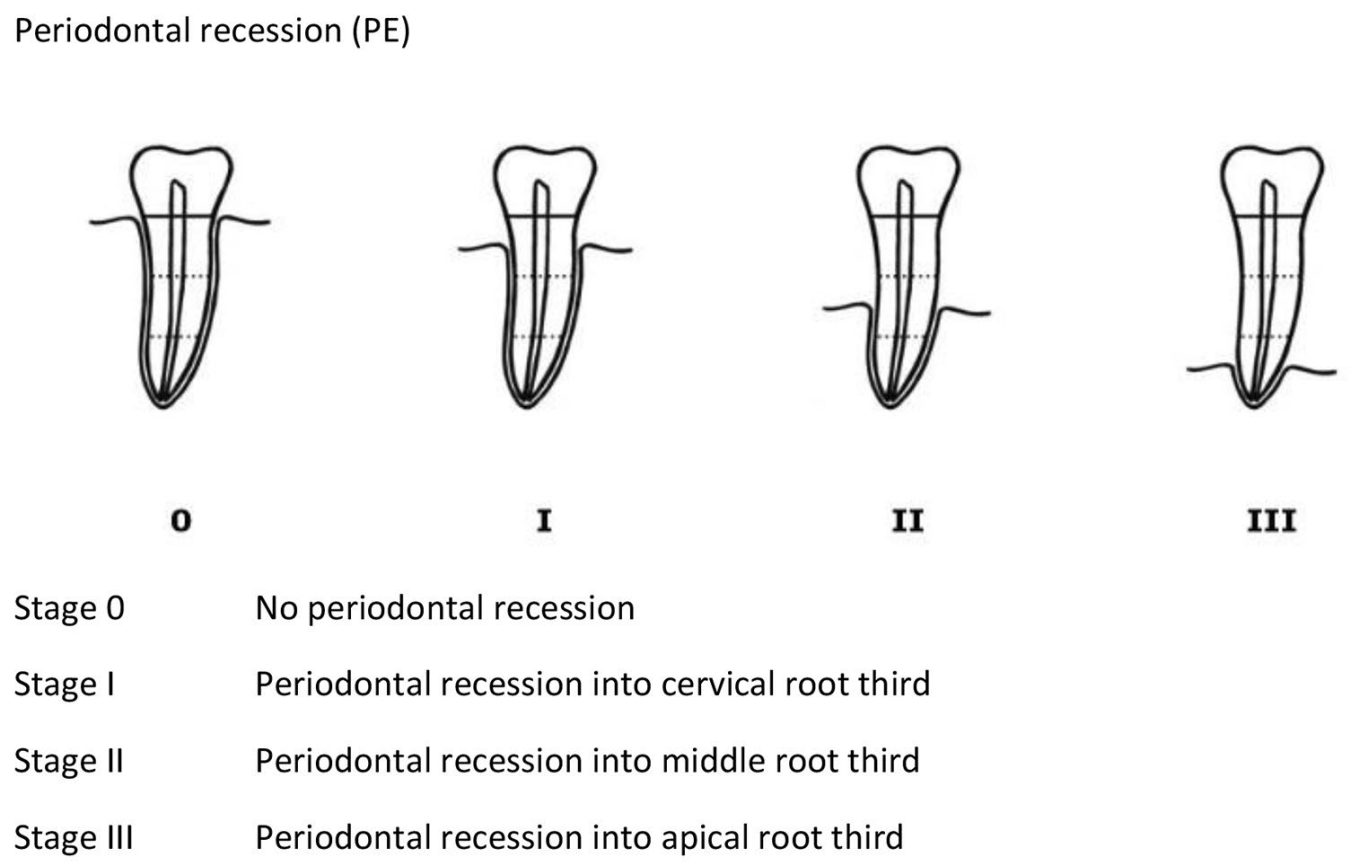
Stage classification to determine degree of periodontal recession [31]

**Fig. 3:**
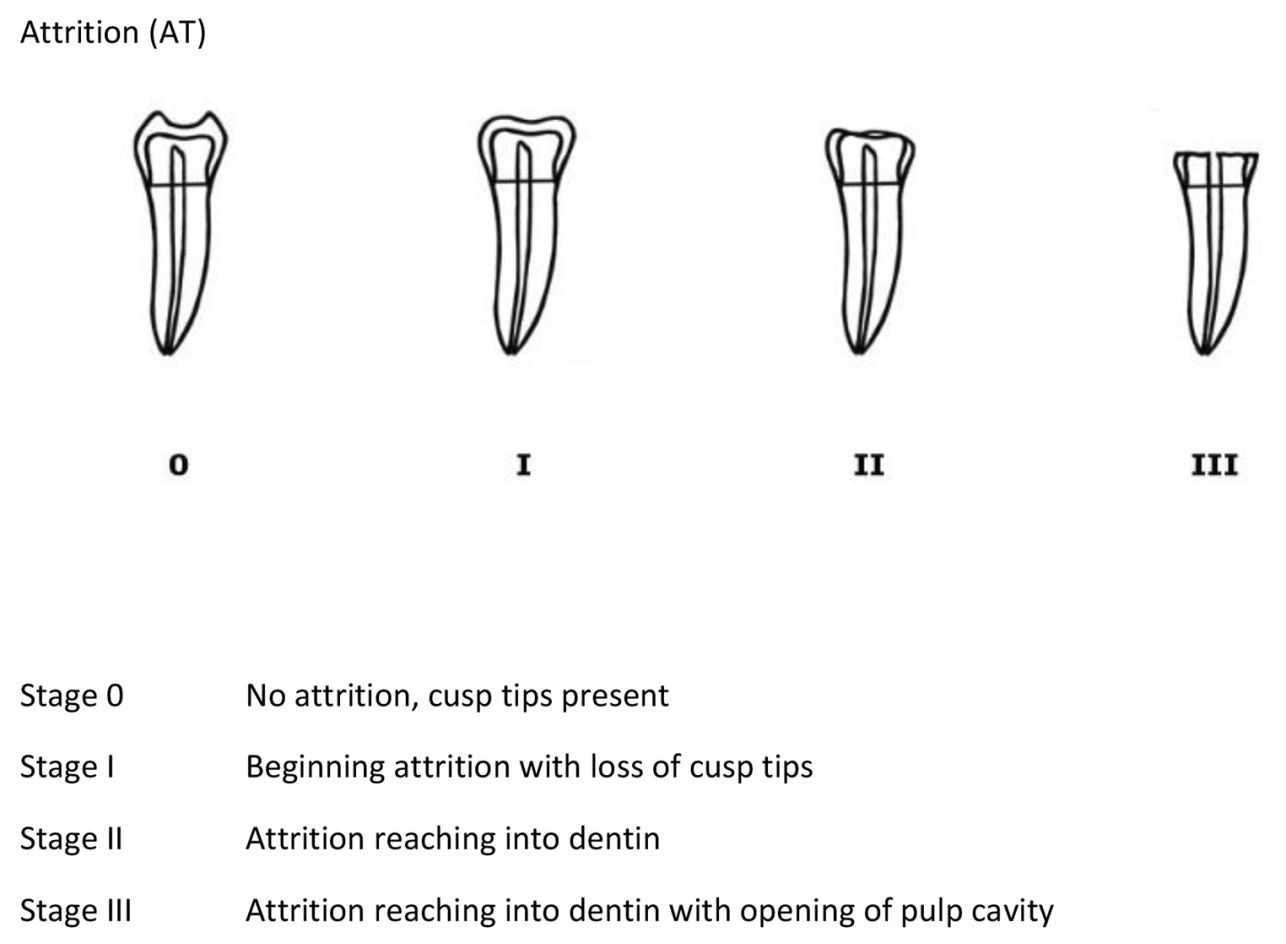
Stage classification to determine degree of attrition [31]

**Fig. 4:**
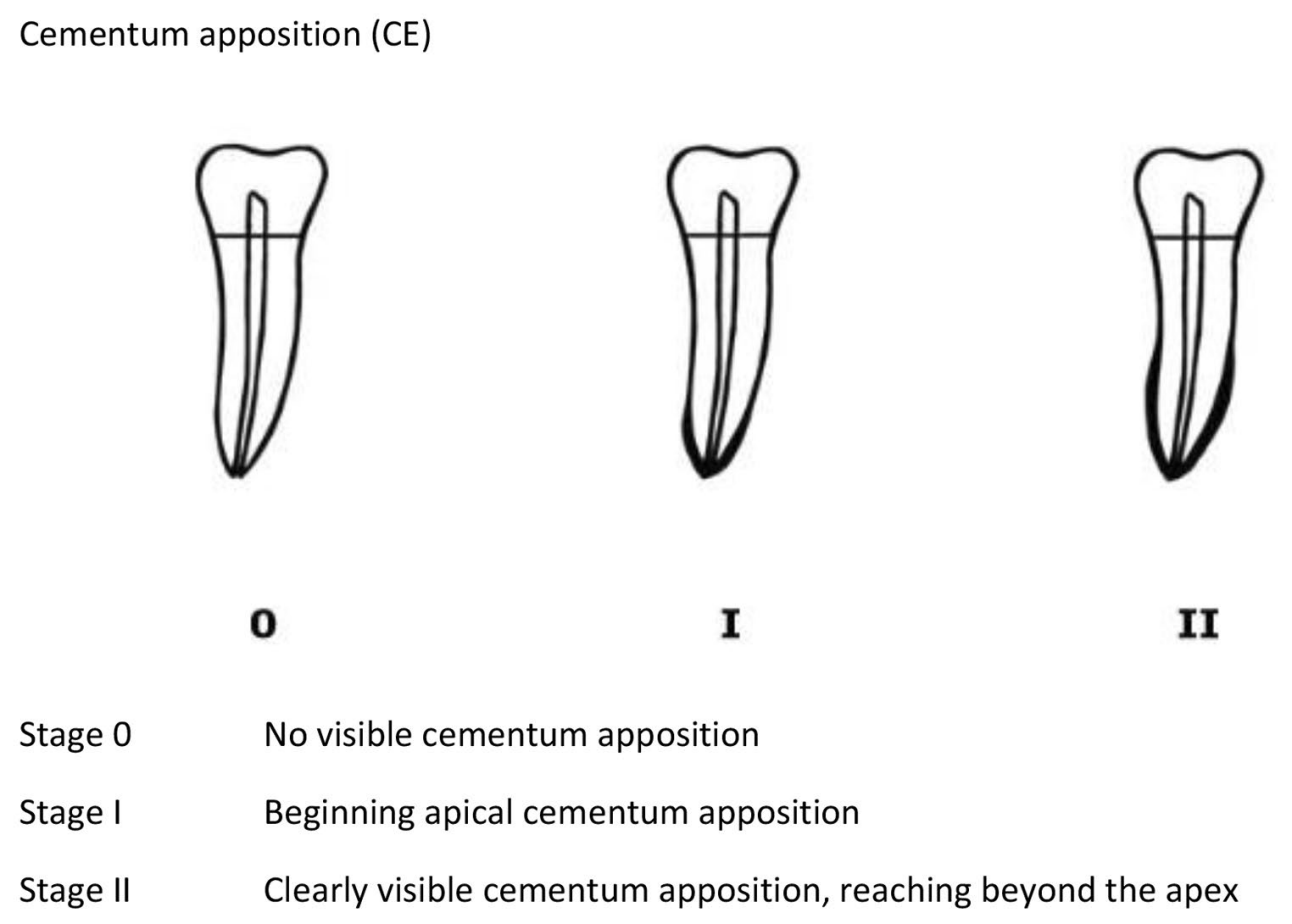
Stage classification to determine degree of cementum apposition [31]

Two board-certified dentists carried out the examinations. One of the two dentists re-evaluated all radiographs at 3-month intervals. The stage classifications according to Olze et al. (2012) [31] were used as shown in Figs. 1, 2, 3 and 4.

The OPGs were analyzed using Synedra Personal View software version 22.0.0 (Synedra Information Technologies GmbH, Innsbruck, Austria) on designated workstations. Both examiners operated under consistent configuration and environmental conditions. The software’s magnification and grey-level adjustment tools were utilized throughout the evaluations.

The two dentists applied the exclusion criteria in the examination, according to Matsikidis [29] (Table 1).

**Table 1:** Exclusion criteria according to Matsikidis [29]

	CL	F	C	*P*	RF	IF	*R*	IM	AE
**AT**			X				X	X	
**SE**	X	X	X	X	X		X		X
**PE**			X				X	X	
**CE**				X	X	X			X

C crowned tooth or bridge abutment, F filling, partial crown or inlay, P post and core restoration, CL carious lesion, RF root filling, IF infected tooth, IM impacted tooth, R retained root, AE apicoectomy, AT attrition, SE secondary dentin formation, PE periodontal recession, CE cementum apposition

The association between individual characteristics and chronological age was examined using multiple regression analysis, with chronological age as the dependent variable. The Gustafson characteristics determined were considered independent variables. The linear regression model was developed in a backward stepwise approach, incorporating the prognosis-relevant influencing variables: attrition, secondary dentin formation, periodontal recession, and cementum apposition. At each step, the significant influencing variable was selected from the remaining variables, with only those having a significance value of ≤ 0.05 included in the model. The coefficient of determination and the standard error of estimate of the regression formula were also calculated.

To assess for potential multicollinearity between the influencing variables, the variance inflation factor (VIF) was calculated for each variable. Multicollinearity, which occurs when one independent variable can be expressed as a linear function of another, was considered critical if the VIF value exceeded 4. In addition, the kappa coefficient for intra- and inter-observer agreement was determined, each with 95% confidence intervals.

Teeth were identified according to the Fédération Dentaire Internationale (FDI) scheme.

## Results

Table 2 shows the composition of the cohort. A total of 1882 OPGs from 934 females and 948 males aged 12.00 (female) to 40.96 (female) years were included.

**Table 2:** Distribution of the sample by chronological age and biological sex

Age (in years)	Female	Male	Total
12	29	30	59
13	28	30	58
14	29	27	56
15	26	27	53
16	30	27	57
17	24	31	55
18	35	29	64
19	30	30	60
20	37	30	67
21	33	26	59
22	30	31	61
23	35	32	67
24	31	33	64
25	35	45	80
26	32	41	73
27	39	38	77
28	31	35	66
29	33	36	69
30	30	35	65
31	33	42	75
32	31	32	63
33	31	31	62
34	38	33	71
35	38	37	75
36	36	28	64
37	32	30	62
38	28	35	63
39	41	34	75
40	29	33	62
**Total**	934	948	1882

Table 3 shows the number of teeth that could not be evaluated and the percentage of teeth that were evaluated. The lowest percentage of teeth was evaluable for the characteristic secondary dentine formation on tooth 45 in males with 84.81%. In contrast, the highest percentage of teeth was evaluable for the characteristic periodontal recession on tooth 34 in females (96.15%). Overall, secondary dentine formation had the fewest evaluable teeth in both sexes, with percentages consistently below 90%. In comparison, all other characteristics achieved evaluation percentages above 90% for all teeth in both sexes.

**Table 3:** Number (n) and percentage of non evaluated and evaluated teeth

Sex	Tooth	Characteristic	Non Evaluated teeth (*n*)	Percentage Non evaluatd	Percentage evaluated
Female	34	SE	104	11.13	88.87
		PE	36	3.85	96.15
		AT	50	5.35	94.65
		CE	42	4.50	95.50
	35	SE	131	14.03	85.97
		PE	65	6.96	93.04
		AT	83	8.89	91.11
		CE	71	7.60	92.40
	44	SE	105	11.24	88.76
		PE	38	4.07	95.93
		AT	51	5.46	94.54
		CE	40	4.28	95.72
	45	SE	139	14.88	85.12
		PE	75	8.03	91.97
		AT	90	9.64	90.36
		CE	76	8.14	91.86
Male	34	SE	112	11.81	88.19
		PE	52	5.49	94.51
		AT	64	6.75	93.25
		CE	47	4.96	95,04
	35	SE	135	14.24	85.76
		PE	72	7.59	92.41
		AT	86	9.07	90.93
		CE	68	7.17	92.83
	44	SE	123	12.97	87.03
		PE	56	5.91	94.09
		AT	67	7.07	92.93
		CE	57	6.01	93.99
	45	SE	144	15.19	84.81
		PE	75	7.91	92.09
		AT	90	9.49	90.51
		CE	76	8.02	91.98

Tables 4 and 5 show the regression formulae for the individual teeth by sex. Correlation with chronological age is consistently higher for males than for females. All R^2^ values are higher for the males than for the corresponding tooth in females. The same is reflected in the standard errors of estimate (SEE): all values for males are below 7 years, while all values for females are above 7 years. It is also noteworthy that while all characteristics could be integrated into the regression formulae for all teeth for the males (*p* < 0.05), this is only the case for tooth 44 for females. For teeth 34, 35 and 45, the characteristic Cementum apposition could not be integrated into the formulae. The reason for this is that the characteristic showed no significant relationship to the dependent variable for these teeth in females (*p* ≥ 0.05).

**Table 4:** Regression equations for the males, coefficients of determination (R²) and standard errors of estimate (SEE) of multiple regression analysis with ages as the dependent variable and dental age changes as independent variables for teeth 34, 35, 44 and 45. **Male**

Tooth	Formula	*R*²	SEE
34	4.0631 + 5.8415*PE + 5.2713*AT + 1.6205*SE + 2.824*CE	0.3492	6.596
35	6.2260 + 5.6510*PE + 4.5287*AT + 1.5254*SE + 1.8698*CE	0.2799	6.93
44	4.7569 + 5.6596*PE + 4.7906*AT + 1.9436*SE + 1.9292*CE	0.3081	6.773
45	3.0585 + 4.4787*PE + 5.7726*AT + 1.8569*SE + 3.2805*CE	0.3382	6.657

**Table 5:** Regression equations for the females, coefficients of determination (R²) and standard errors of estimate (SEE) of multiple regression analysis with ages as the dependent variable and dental age changes as the independent variables for teeth 34, 35, 44 and 45. **Female**

Tooth	Formula	*R*²	SEE
34	7.6739 + 4.5040*PE + 5.8362*AT + 1.8493*SE	0.2458	7.202
35	6.8866 + 5.1977*PE + 5.6223*AT + 1.9380*SE	0.2225	7.319
44	5.3323 + 4.7451*PE + 5.5017*AT + 2.2508*SE + 1.3243*CE	0.2352	7.261
45	5.3828 + 4.6529*PE + 5.5112*AT + 2.5539*SE	0.2579	7.119

The was no evidence of multicollinearity, as all Variance Inflation factor values (VIFs) were below the critical threshold of 4 (Table 6).

**Table 6:** Variance inflation factor values (VIF) for teeth 34, 35, 44 and 45. Presented here as the highest value calculated from the individual characteristics

Tooth	Males	Females
34	1.04	1.04
35	1.03	1.03
44	1.03	1.03
45	1.05	1.05

The data for intra- and inter-observer agreement are presented in Tables 7 and 8. Kappa values ranged from 0.092 for the characteristic cementum apposition on tooth 34 to 0.98 for the characteristic attrition on tooth 44. It is particularly notable that the observer agreement for the cementum apposition feature is very low, especially for the first premolars. The observer agreement for the same feature on the second premolars is in the range of the agreements for the other features. Overall, the kappa values for inter-rater agreement are lower than those for intra-rater agreement across all teeth and characteristics. If the outlier values for the characteristic cement apposition at the first premolars are ignored, the kappa values for the interobserver agreement range from 0.28 to 0.59. The kappa values for the intra-observer agreement then range from 0.58 to 0.98. According to Landis and Koch, these values range from “fair” to “moderate” inter-observer agreement. The values for intra-observer agreement range from “moderate” to “almost perfect” [44]. The low values for both observer agreements for the cementum apposition characteristic on the first premolars are in the “slight” range [44].

**Table 7:** Inter-rater reliability [kappa] for the characteristics. CI: confidence interval

Tooth	Characteristic	kappa	Upper CI	Lower CI
34	AT	0.28	0.24	0.33
34	CE	0.092	0.05	0.14
34	PE	0.53	0.48	0.57
34	SE	0.51	0.48	0.56
35	AT	0.36	0.32	0.41
35	CE	0.13	0.07	0.18
35	PE	0.43	0.38	0.47
35	SE	0.45	0.41	0.5
44	AT	0.43	0.39	0.47
44	CE	0.44	0.38	0.49
44	PE	0.59	0.55	0.63
44	SE	0.53	0.5	0.57
45	AT	0.44	0.4	0.48
45	CE	0.35	0.3	0.42
45	PE	0.55	0.51	0.58
45	SE	0.5	0.45	0.55

**Table 8:** Intra-rater reliability [kappa] for the characteristics. CI: confidence interval

Tooth	Characteristic	kappa	Upper CI	Lower CI
34	AT	0.74	0.71	0.77
34	CE	0.13	0.08	0.2
34	PE	0.61	0.57	0.65
34	SE	0.66	0.62	0.69
35	AT	0.78	0.75	0.81
35	CE	0.12	0.07	0.19
35	PE	0.59	0.55	0.64
35	SE	0.58	0.54	0.62
44	AT	0.98	0.97	0.99
44	CE	0.77	0.72	0.81
44	PE	0.71	0.67	0.75
44	SE	0.68	0.65	0.71
45	AT	0.98	0.97	0.99
45	CE	0.73	0.67	0.79
45	PE	0.71	0.67	0.75
45	SE	0.59	0.55	0.63

Due to the peculiarities in the cementum apposition characteristic, the primary evaluations are analyzed further. It is important to note that a stage 0 was identified for this characteristic on tooth 34 in a total of 1562 cases across both sexes by one examiner, representing 83.0% of the total cohort. Additionally, 212 cases (11.26%) were classified as stage I. For tooth 44, the values for a determination of stage 0 are 1504 cases or 79.91%. Stage I was classified for this tooth in 257 cases (13.66%). The second examiner determined a stage 0 for cement apposition on tooth 34 in 1444 cases (76.73%). For tooth 44, these values are 1429 and 75.93% respectively. Stage I for cementum apposition was determined by the second examiner for teeth 34 and 44 in 336 (17.85%) and 340 (18.07%) cases, respectively.

Stage 0 covers the entire age range of the study. For example, the oldest person in the cohort (female, 40.96 years) also shows a stage 0 for cement apposition on all teeth in both examination rounds of examiner 1. In contrast, examiner 2 attested a stage I for this female on teeth 34 and 45.

The cases in which both examiners assigned a stage II for cement apposition on the first premolars are almost congruent (age range: 27.04–40.44 years).

## Discussion

In the current study, regression formulae were developed in which the characteristic values of the individual degenerative Gustafson tooth features are combined to produce an age estimate. Therefore, the method’s strength is not based on using a single characteristic for age estimation, but on the combination of different characteristics using an age-associated overall degeneration of hard and soft dental tissues.

Our study is an integral part of the evolutionary development of the method and the resulting research questions. The method was first developed by Olze et al. (2012), who transferred the Gustafson’s criteria to the OPG and presented regression formulae for practical use for the first time [31]. In 2017, Timme et al. validated this method on a larger population and a larger age range [32]. Subsequently, Si et al. (2019) evaluated the method in a cohort from northern China [33]. Thus, a comparison of the results of the present study with these previous studies is required (Table 9).

**Table 9:** Comparison of the studies investigating Gustafsons criteria using the Olze et al. (2019) staging scales. For better comparability, the values of the present study are included again, although they are already listed in other tables. VIF: Variance inflation factor; R^2^: coefficients of determination; SEE: standard errors of estimate in years; - : no information given

	Olze et al. 2012 [31]	Timme et al. 2017 [45]	Si et al. 2019 [33]	Present study
General characteristics				
Individuals (n) Total	1299	1245 (as separate age group rating)	1300	1882
Females	650	606	650	934
Males	649	639	650	948
Age groups [years]	15–40	15–40 (as separate rating)	15–40	12–40
Reliability of participants’ age information	High	High	High	High
Geographic origin of the study population	Germany	Germany	China (northern China)	South Africa (Pretoria)
Ethnicity	White	White	Asian	Black
Study design	retrospective	retrospective	retrospective	retrospective
Staging scales	Olze et al. 2012	Olze et al. 2012	Olze et al. 2012	Olze et al. 2012
Generation period of X-rays	1987–2008	1985–2011	2011–2014	2011–2022
Special exclusion criteria	Matsikidis	Matsikidis	Matsikidis	Matsikidis
Results				
Intra-rater agreement [kappa]	-	0.78–0.92	0.639–0.893	0.13–0.98
Inter-rater agreement [kappa]	-	0.38–0.75	0.285–0.587	0.092–0.59
VIF	1.16–1.42	all < 4	-	1.03–1.05
evaluated teeth [%]	44.77–60.40 By sex and tooth	20.10-59.12 By sex and tooth	70.00-82.93 By sex, tooth and age group	84.81–96.15 By sex, tooth and characteristic
Regression equations, sex; tooth R^2^ | SEE				
Males; 34	0.48 | 5.4	0.49 | 5.1	0.64 | 4.54	0.35 | 6.6
Males; 35	0.49 | 5.4	0.59 | 4.64	0.7 | 4.31	0.28 | 6.93
Males; 44	0.52 |5.5	0.47 | 5.17	0.65 | 4.63	0.31 |6.78
Males; 45	0.53 | 5.3	0.51 | 4.93	0.68 | 4.53	0.34 | 6.66
Females; 34	0.44 | 5.7	0.49 | 5.14	0.64 | 4.29	0.25 | 7.20
Females; 35	0.47 | 5.5	0.54 | 4.71	0.68 | 4.75	0.22 | 7.32
Females; 44	0.43 | 5.7	0.5 | 5.05	0.66 | 4.75	0.24 | 7.26
Females; 45	0.48 | 5.4	0.55 | 4.68	0.69 | 4.64	0.26 | 7.12

In direct comparison with these studies (Table 9), the slightly different age range of the cohort is imposing. It was decided to lower the minimum age of the cohort from 15 to 12 years compared to the previous studies using the method, as there are indications in the literature that the development of the third molars and thus the entire tooth development in a black population from the Sub-Saharan region may be completed earlier [45, 46]. We are doing justice to this aspect by lowering the minimum age in the cohort, as the Gustafson’s criteria become relevant when tooth development is complete and the age information that can be derived from this is limited.

It is noteworthy that the values for observer agreements in the current study are in part significantly lower than in the other studies (Table 9). As mentioned in the results section, the low values for both intra- and inter-observer agreement are mainly due to the values for the cementum apposition characteristic on the first premolars. If these outlier values are not taken into account, the values of the present study are within the range of the values published by Si et al. [33]. If the characteristic cementum apposition on the first premolars is further analyzed, it is noticeable that a stage 0 or I was found in over 90% of the determinations, with stage 0 alone regularly accounting for over 75% of the cases. The few cases in which a stage II was assigned are almost congruent. This shows that the vast majority of the cohort had no cementum apposition. In addition, the low values for observer agreement result from differences in the assignment of a stage 0 or I, i.e. specifically whether a finding was assessed as “no cementum apposition” or as “beginning” or minimal cementum apposition (Fig. 4). Si et al. also concluded in 2019 that the precision of the age estimate could be compromised by the more difficult determination of the cementum apposition feature [33]. From our point of view, it is critical that Olze et al. (2012) did not provide a more specific formulation for differentiating between the two stages. For the future, it would therefore be desirable to find a concrete definition, ideally with concrete landmarks, for the differentiation between stage 0 and I for the cementum apposition.

The overall low level of cementum apposition across all age groups in the cohort also explains why, in contrast to the other studies (Table 9), the characteristic was not included in all regression formulae. In fact, the characteristic is included in all formulae for males, but only in the formula for tooth 44 for females. This could actually be a specific finding of the analyzed cohort. Future studies should investigate whether the cementum apposition in Sub-Saharan Africans actually appears differently than in other populations or whether there is a sex difference for this characteristic in this particular population.

A fundamental shortcoming of the characteristic is that cementum cannot be appreciated radiographically, as the contrast between cement and dentine is minimal [47]. This means that cementum apposition as such can only be determined by an apical change in the shape of the tooth (Fig. 4), which requires advanced cementum apposition, but also a great deal of experience on the part of the examiner and perfect image quality. The characteristic of cementum apposition can therefore be regarded as particularly challenging in several respects.

The percentage of evaluable teeth in our study is significantly higher than in other previous studies (Table 9). This result is mainly due to a pre-selection of the images when collecting the images from the clinic’s archive. In particular, only orthopantomograms in which the mandibular premolars were present were included in the study. This approach was chosen so that as many cases as possible could ultimately be included in the statistical analyses and so that the results would be as reliable as possible on a broad basis. In contrast, in the other studies (Table 9), many cases that could not be analyzed due to missing teeth, particularly in the study by Timme et al. from 2017 [32]. The reason why in our study the lowest values for the evaluability of the teeth were consistently found for the secondary dentine formation characteristic (Table 3) is due to the fact that the Matsikidis exclusion criteria for this characteristic, compared to the other characteristics, give the most reasons for exclusion (Table 1) [29].

The values for the coefficients of determination and the standard errors of estimate for the regression formulae are consistently worse for females than for males in the present study (Tables 4 and 5). This is a new finding of a sex difference not observed in the previous studies (Table 9). Based on these results, it can be concluded that the Gustafson’s criteria for black females from the Sub-Saharan region are unsuitable for proof of completion of relevant age limits due to the low precision in age estimation. Whether this is a general phenomenon in the population, or a specific finding of this study cohort must be clarified in future studies. Until this question has been finally addressed, the regression formulae should not be used as the exclusive criterion for age estimation in relevant female groups.

If the regression equations of the different studies are compared with each other (Table 9), it is evident that the values for the coefficients of determination and the standard errors of estimate in the present study are lower and, in some cases, substantially lower than the values in the other studies. This indicates that the correlation of the Gustafson’s criteria with chronological age is lower in the study population than in the comparison populations. The reason for this observation cannot be conclusively clarified. On the one hand, an influence of ethnicity as such on the expression of the Gustafson’s criteria could be present. However, external factors must be considered as factors that can influence the characteristics. These may include diet, habits such as bruxism or general health such as periodontitis [38]. However, indirect influences must also considered, for example the number of (remaining) teeth in the mouth is important for the characteristics of attrition [38]. All in all, life circumstances influence the Gustafson’s criteria, with living conditions represented by socio-economic status, for example [48]. Compared to the studies by Olze et al. and Timme et al. [31, 32] (Table 9), the socio-economic status of the current cohort is lower. It is therefore also conceivable that the socio-economic status or living conditions explain the differences between the studies (Table 9) rather than the influence of ethnicity on the characteristics. There is a great need for future research in this regard.

Another study on this topic was conducted by Dai et al. in 2024 [49]. The authors examined the Gustafson’s criteria in a southwest Chinese cohort of 851 individuals, 402 females and 449 males. The study is not directly comparable with the studies in Table 9, as Dai et al. did not analyze the characteristic of cementum apposition. The reason for this approach was explicitly the poor observer agreement for this characteristic in the study by Si et al. from 2019 [33, 49]. For the coefficients of determination and the standard errors of estimate, Dai et al. were able to achieve values (R^2^ = 0.260–0.565; SEE = 5.560–6.719) with their regression formulae that are below the values of Si et al. for a different Chinese population. The values of Dai et al. are more in the range of the values of the present study. However, the study by Dai et al. is of particular interest for its incorporation of an additional analysis of the examiners’ results, which were not only subjected to the familiar multiple regression analysis. Dai et al. also tested various machine learning models [49]. Some machine learning models outperformed the multiple regression analyses. The best results were obtained using partial least squares regression for males and support vector regression for females. The approach of Dai et al. can be seen as very forward-looking. It can be assumed that technologies such as machine learning or artificial intelligence (AI) will be increasingly used in forensic age estimation in the future. Other publications also point in this direction [50–57]. Nevertheless, we also consider it important to familiarize with the fundamentals of this field of research before using advanced technology in order to better understand the results of automated procedures. Our study should also be seen in the context of this necessary basic research.

The study by Dai et al. raises questions about the suitability of linear regression for forensic age estimation [49]. While linear regression is typically appropriate for continuous variables [58, 59], the age estimation characteristics evaluated in this study are ordinally scaled. A Bayesian approach was proposed for the multivariable analysis of ordinal outcome variables in forensic age assessment [60], but it did not enhance the accuracy of estimations compared to linear regression [60]. Timme et al. and Si et al. also argued from this perspective when they used the regression models in their studies [32, 33]. Additionally, ordinal regression or rescaled linear regression are alternative methods for analyzing such data [61, 62]. However, these alternative methods were not employed in the present study to maintain consistency with existing literature (see Table 9).

Overall, it is important to note that using multiple regression for ordinally scaled characteristics represents a compromise that may not be suitable in all cases, especially when the continuity of variables cannot be resolved with certainty. The use of categorical variables in regression analyses presents unique challenges, but in this context, linear regression remains an accepted and validated method.

In 2021, Dezem et al. conducted an empirical investigation into the regression formulae proposed by Olze et al. (2012) and Timme et al. (2017) [63]. The authors applied the regression formulae from the existing literature to a collective of 503 individuals from Brazil, aged between 20 and 70 years. Additionally, a subgroup analysis was conducted for the 15–40 age group. In their analyses, the authors identified lower values for the correlation with age than those initially reported in the formulae. Dezem et al. conclude that the formulae for age estimation in Brazilian populations are unsuitable and that population-specific formulae are necessary [63]. We concur with this statement and conclude that the regression formulae should currently only be employed in populations as identical as possible to the validation population. Additionally, it is imperative to consider the potential for population-specific formulae, particularly given the possibility of differing formulae emerging depending on socio-economic status, contingent on advancing of research in this field. A primary focus for the future research may be that reference studies for degenerative dental characteristics are not required for specific (ethnic) populations, but rather for socio-economic status groups. For this purpose, samples with identical ethnicity and different socio-economic status as well as samples with identical socio-economic status and different ethnicity should be analyzed comparatively.

## Conclusions

The correlation between the Gustafson’s criteria and age using OPGs was consistently lower for female subjects in the study population compared to male subjects. Overall, these correlations were lower to those reported in the literature for other populations. Therefore, based on these data, we recommended that the Gustafson’s criteria should not be used as the primary age estimator for black individuals from Sub-Saharan Africa, particularly females. Future studies should investigate whether ethnicity or socioeconomic status plays a significant role on the characteristics in question. Given the low observer agreement for cementum apposition, future research should aim to develop a more selective staging scale for this characteristic.

## Data Availability

The datasets generated during the current study are available from the corresponding author upon reasonable request.
